# Clinicopathological, molecular, and prognostic features of colorectal carcinomas with *KRAS* c.34G>T (p.G12C) mutation

**DOI:** 10.1111/cas.16262

**Published:** 2024-07-22

**Authors:** Satoko Ugai, Qian Yao, Yasutoshi Takashima, Yuxue Zhong, Kosuke Matsuda, Hidetaka Kawamura, Yu Imamura, Kazuo Okadome, Kosuke Mima, Kota Arima, Keisuke Kosumi, Mingyang Song, Jeffrey A. Meyerhardt, Marios Giannakis, Jonathan A. Nowak, Tomotaka Ugai, Shuji Ogino

**Affiliations:** ^1^ Program in MPE Molecular Pathological Epidemiology, Department of Pathology Brigham and Women's Hospital, and Harvard Medical School Boston Massachusetts USA; ^2^ Department of Epidemiology Harvard T.H. Chan School of Public Health Boston Massachusetts USA; ^3^ Department of Medical Oncology Dana‐Farber Cancer Institute and Harvard Medical Boston Massachusetts USA; ^4^ Department of Surgery Fukushima Medical University Fukushima Japan; ^5^ Department of Esophageal Surgery The Cancer Institute Hospital of the Japanese Foundation of Cancer Research Tokyo Japan; ^6^ Department of Gastroenterological Surgery, Graduate School of Medical Sciences Kumamoto University Kumamoto Japan; ^7^ Department of Nutrition Harvard T.H. Chan School of Public Health Boston Massachusetts USA; ^8^ Clinical and Translational Epidemiology Unit Massachusetts General Hospital and Harvard Medical School Boston Massachusetts USA; ^9^ Division of Gastroenterology Massachusetts General Hospital Boston Massachusetts USA; ^10^ Broad Institute of MIT and Harvard Cambridge Massachusetts USA; ^11^ Department of Medicine Brigham and Women's Hospital and Harvard Medical School Boston Massachusetts USA; ^12^ Cancer Immunology Program Dana‐Farber/Harvard Cancer Center Boston Massachusetts USA; ^13^ Tokyo Medical and Dental University (Institute of Science Tokyo) Tokyo Japan

**Keywords:** adagrasib, colorectal tumor, CpG island methylator phenotype, *KRAS*, sotorasib

## Abstract

Evidence indicates that combinations of anti‐EGFR antibodies and KRAS p.G12C (c.34G>T) inhibitors can be an effective treatment strategy for advanced colorectal cancer. We hypothesized that *KRAS* c.34G>T (p.G12C)‐mutated colorectal carcinoma might be a distinct tumor subtype. We utilized a prospective cohort incident tumor biobank (including 1347 colorectal carcinomas) and detected *KRAS* c.34G>T (p.G12C) mutation in 43 cases (3.2%) and other *KRAS* mutations (in codon 12, 13, 61, or 146) in 467 cases (35%). The CpG island methylator phenotype (CIMP)‐low prevalence was similarly higher in *KRAS* c.34G>T mutants (52%) and other *KRAS* mutants (49%) than in *KRAS*‐wild‐type tumors (31%). *KRAS* c.34G>T mutants showed higher CIMP‐high prevalence (14%) and lower CIMP‐negative prevalence (33%) compared with other *KRAS* mutants (6% and 45%, respectively; *p* = 0.0036). Similar to other *KRAS* mutants, *KRAS* c.34G>T‐mutated tumors were associated with cecal location, non‐microsatellite instability (MSI)‐high status, *BRAF* wild type, and *PIK3CA* mutation when compared with *KRAS*‐wild‐type tumors. Compared with *BRAF*‐mutated tumors, *KRAS* c.34G>T mutants showed more frequent LINE‐1 hypomethylation, a biomarker for early‐onset colorectal carcinoma. *KRAS* c.34G>T mutants were not associated with other features, including the tumor tissue abundance of *Fusobacterium nucleatum* (*F. animalis*), *pks* ^+^ *Escherichia coli*, *Bifidobacterium*, or (enterotoxigenic) *Bacteroides fragilis*. Among 1122 *BRAF*‐wild‐type colorectal carcinomas, compared with *KRAS*‐wild‐type tumors, multivariable‐adjusted colorectal cancer‐specific mortality hazard ratios (95% confidence interval) were 1.82 (1.05–3.17) in *KRAS* c.34G>T (p.G12C)‐mutated tumors (*p* = 0.035) and 1.57 (1.22–2.02) in other *KRAS*‐mutated tumors (*p* = 0.0004). Our study provides novel evidence for clinical and tumor characteristics of *KRAS* c.34G>T (p.G12C)‐mutated colorectal carcinoma.

## Graphical abstract

The CpG island methylator phenotype (CIMP)‐low prevalence was similarly higher in *KRAS* c.34G>T mutants and other *KRAS* mutants than in *KRAS*‐wild‐type tumors. *KRAS* c.34G>T mutants showed higher CIMP‐high prevalence and lower CIMP‐negative prevalence compared with other *KRAS* mutants. Similar to other *KRAS* mutants, *KRAS* c.34G>T‐mutated tumors were associated with cecal location, non‐microsatellite instability (MSI)‐high status, *BRAF* wild type, and *PIK3CA* mutation when compared with *KRAS*‐wild‐type tumors.

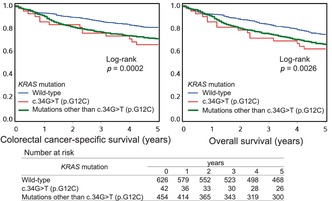

**Abbreviations**
AJCC: American Joint Committee on Cancer
CI: confidence interval
CIMP: CpG island methylator phenotype
FFPE: formalin‐fixed paraffin‐embedded
HGNC: Human Genome Organisation Gene Nomenclature Committee
HR: hazard ratio
LINE‐1: long interspersed nucleotide element‐1
MSI: microsatellite instability
PCITB: prospective cohort incident tumor biobank
PCR: polymerase chain reaction

## 1 INTRODUCTION

Colorectal cancer is a complex disease with a variety of somatic mutations in oncogenes and tumor suppressor genes.^1^, ^2^ *KRAS* is a proto‐oncogene related to the RAS/mitogen‐activated protein kinase (MAPK) pathway. Approximately 40% of colorectal cancers harbor *KRAS* mutations, most commonly in codons 12, 13, and 61.^3^ *KRAS*‐mutated tumors are insensitive to treatment with anti‐EGFR antibodies, including cetuximab and panitumumab.^4^, ^5^, ^6^

Of *KRAS* oncogenic mutations, *KRAS* c.34G>T (p.G12C) mutation occurs in 3%–4% of colorectal cancer patients.^7^ Compared with other *KRAS* mutations, *KRAS* c.34G>T (p.G12C) mutation has been associated with worse survival in advanced colorectal cancer patients who received conventional chemotherapy.^8^, ^9^, ^10^ Recently, novel specific KRAS c.34G>T (p.G12C) protein inhibitors have shown promising results for better survival in non‐small cell lung cancer patients with *KRAS* c.34G>T (p.G12C)‐mutated tumors.^11^, ^12^ Clinical studies have indicated that a combination of anti‐EGFR targeted therapy and *KRAS* c.34G>T (p.G12C) inhibitor could be an effective treatment strategy in *KRAS* c.34G>T (p.G12C)‐mutated colorectal cancer.^13^, ^14^ However, unique features of *KRAS* c.34G>T‐mutated colorectal carcinoma (compared with *KRAS*‐wild‐type and other *KRAS*‐mutated tumors) need to be further elucidated.

Therefore, we tested a hypothesis that clinical and tumor characteristics of *KRAS* c.34G>T (p.G12C)‐mutated tumors might differ from those of other *KRAS*‐mutated tumors and *KRAS*‐wild‐type tumors. To test our hypothesis, we used the integrated database of colorectal carcinoma patients within two United States‐wide prospective cohort studies.

## 2 MATERIALS AND METHODS

### 2.1 Study population

We used two United States‐wide prospective cohort studies: the Nurses' Health Study including 121,701 women aged 30–55 years at enrollment in 1976 and the Health Professionals Follow‐up Study including 51,529 men aged 40–75 years at enrollment in 1986.^15^, ^16^ Every 2 years, cohort participants have been sent follow‐up questionnaires to identify newly diagnosed cancers in themselves and their first‐degree relatives. The response rate exceeded 90% for each follow‐up questionnaire cycle in both cohorts. The National Death Index was used to ascertain death and identify participants with unreported lethal colorectal cancer. Study physicians, blinded to exposure data, reviewed documents of identified colorectal cancer cases to confirm the diagnosis, record clinical and tumor characteristics, and determine the cause of death if it had occurred. Formalin‐fixed paraffin‐embedded (FFPE) tissue blocks were collected from hospitals where surgeons resected colorectal cancers of cohort participants, which led to the creation of the prospective cohort incident tumor biobank (PCITB). A single pathologist (S. Ogino), unaware of other data, examined hematoxylin and eosin‐stained sections of all cases, and recorded pathological features including tumor differentiation.

**FIGURE 1:**
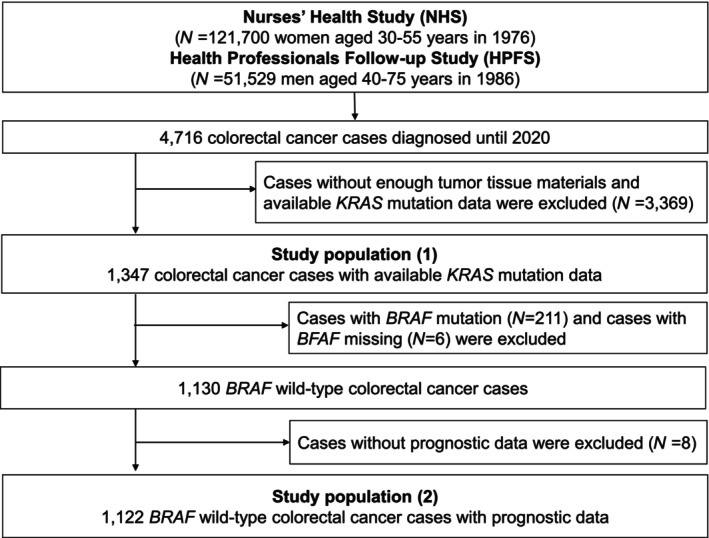
Flow diagram of the study participants.

Among 4716 incident colorectal cancer cases that had occurred in the Nurses' Health Study and the Health Professionals Follow‐up Study up to 2014, the current study included 1347 colorectal carcinoma cases with enough tumor tissue materials and available *KRAS* mutation data in the PCITB (Figure 1). When assessing the prognostic effect of *KRAS* mutations, it is important to assess their prognostic effect independent of *BRAF* c.1799T>A (p.V600E) mutation because this *BRAF* mutation has been associated with poorer prognosis and strongly inversely associated with *KRAS* mutation.^17^, ^18^ There were only seven patients with *KRAS* mutations (with no c.34G>T mutant) among 211 *BRAF*‐mutated tumor cases, which made it impossible to robustly assess the prognostic role of *KRAS* mutations in the *BRAF*‐mutated tumor stratum. For patient survival analyses, we excluded 211 *BRAF* c.1799T>A‐mutated cases, six cases with missing *BRAF* mutation status, and eight cases with missing survival data, leaving 1122 tumors, which were designated as “*BRAF* wild type” (though we did not analyze other less frequent *BRAF* mutations that can alter protein functions) (Figure 1).

The study protocol was approved by the institutional review boards of the Brigham and Women's Hospital and Harvard T.H. Chan School of Public Health, and those of participating registries as required.

### 2.2 Tumor tissue analyses

Genomic DNA was extracted from FFPE tumor tissue (and paired normal tissue) sections, using guide slides of tissue sections with marked tumor areas. Polymerase chain reaction (PCR) and pyrosequencing were used to detect mutations in *KRAS* (codons 12, 13, 61, and 146),^19^, ^20^ *BRAF* (codon 600), and *PIK3CA* (exons 9 and 20)^21^ as previously described.^22^ MSI analysis was performed using 10 microsatellite markers (D2S123, D5S346, D17S250, BAT25, BAT26, BAT40, D18S55, D18S56, D18S67, and D18S487). MSI‐high was defined as instability in ≥30% of the markers.^23^ Using validated bisulfite DNA treatment and real‐time PCR (MethyLight),^24^ we quantified DNA methylation in eight CpG island methylator phenotype (CIMP)‐specific promoters (*CACNA1G*, *CDKN2A* [p16], *CRABP1*, *IGF2*, *MLH1*, *NEUROG1*, *RUNX3*, and *SOCS1*).^25^, ^26^ CIMP‐high, CIMP‐low, and CIMP‐negative statuses were defined as ≥5/8 methylated markers, 1/8 to 4/8 methylated markers, and 0/8 methylated markers, respectively, using the 8‐marker CIMP panel. These criteria were modified ones from the original ones, considering the prevalence of both *KRAS* and *BRAF* mutations.^25^ To accurately quantify long interspersed nucleotide element 1 (LINE‐1) methylation levels, bisulfite pyrosequencing was used as previously described.^27^, ^28^ To measure the relative DNA amount of *Fusobacterium nucleatum* (now named, *F. animalis*), *pks* ^+^ *Escherichia coli*, *Bacteroides fragilis*, enterotoxigenic *B. fragilis* (ETBF), and *Bifidobacterium* in tumor tissue, we performed quantitative PCR with primers targeting the *nusG* gene of *F. nucleatum*, the *clbB* gene of *pks* ^+^ *E. coli*, the *RecA* and *bft* genes of *B. fragilis*, and the 16S ribosomal RNA gene DNA sequences of *Bifidobacterium*, as previously described.^29^, ^30^, ^31^, ^32^

### 2.3 Statistical analysis

The chi‐square test or Fisher's exact test was used to compare categorical data, while an analysis of variance was applied to continuous variables (age and LINE‐1 methylation). To compare ordinal variables, including the American Joint Committee on Cancer (AJCC) disease stage, and tumor location, the Spearman correlation test was performed. The Kaplan–Meier method and log‐rank test were used to estimate survival distribution according to *KRAS* mutation status. Cases were observed until death, or January 1, 2020, whichever came first. For analyses of colorectal cancer‐specific mortality, deaths as a result of other causes were censored. Cox proportional hazards regression models were used to compute the mortality hazard ratio (HR) for patient groups. The multivariate Cox model initially included the following variables: sex, age at colorectal cancer diagnosis (continuous), year of diagnosis (prior to 1995 vs. 1996–2000 vs. 2001–2014), family history of colorectal cancer in any first‐degree relative (present vs. absent), tumor location (cecum vs. ascending/transverse colon vs. distal colon vs. rectum), tumor differentiation (well to moderate vs. poor), CIMP (high vs. low/negative), *PIK3CA* mutation (mutant vs. wild‐type), and LINE‐1 methylation (continuous). A backward elimination was performed with a threshold of *p* = 0.05 to limit the number of covariates and avoid overfitting of the final multivariable models. Cases with missing information for any of the categorical covariates (family history of colorectal cancer [0.4%], tumor differentiation [0.6%], MSI [1.9%], CIMP [7.1%], and *PIK3CA* [7.6%]) were included in the majority category of the given covariate to avoid overfitting. We made a missing indicator variable for cases of missing tumor location information (0.5%). Eventually, all tumor location variables were eliminated by the selection procedure. The proportional hazards assumption was assessed by a time‐varying covariate (i.e., the cross‐product of the *KRAS* mutation status and survival time). The assumption was satisfied for 5‐year survival analyses. Therefore, we analyzed the prognostic association of *KRAS* mutation status with censoring of all 5‐year survivors in the survival analysis. All statistical analyses were performed using SAS version 9.2 (SAS Institute). All *p*‐values were two‐sided. We used the two‐sided alpha level of 0.005 as recommended by the expert panel^33^ and regarded *p*‐values between 0.005 and 0.05 as suggestive evidence.

## 3 RESULTS

Among 1347 colorectal carcinoma cases with *KRAS* mutation data in the two United States‐wide prospective cohort studies, we detected *KRAS* c.34G>T (p.G12C) mutation in 43 cases (3.2%) and other *KRAS* mutations (i.e., *KRAS* mutations that were not c.34G>T) in 467 cases (35%). Table 1 shows the characteristics of study participants according to *KRAS* mutation status.

**TABLE 1:** Clinicopathological, molecular, and microbial characteristics according to *KRAS* mutation status in 1347 colorectal cancer cases.

Characteristics^a^	All cases	*KRAS*			*p* value (c.34G>T vs. wild‐type)^b^	*p* value (c.34G>T vs. mutations other than c.34G>T)^b^
		Wild‐type	Mutations other than c.34G>T (p.G12C)	c.34G>T (p.G12C)		
	(*N* = 1347)	(*N* = 837)	(*N* = 467)	(*N* = 43)		
Sex						
Male (HPFS)	601 (45%)	345 (41%)	235 (50%)	21 (49%)	0.32	0.85
Female (NHS)	746 (55%)	492 (59%)	232 (50%)	22 (51%)		
Mean age ± SD (years)	69.0 ± 10.1	69.1 ± 8.9	69.0 ± 8.9	70.3 ± 8.9	0.39	0.29
Cumulative pack‐years smoked						
0	528 (41%)	334 (41%)	174 (40%)	20 (50%)	0.64	0.36
1–19	313 (24%)	194 (24%)	109 (25%)	10 (25%)		
20–39	225 (17%)	140 (17%)	73 (19%)	4 (10%)		
≥40	224 (17%)	145 (18%)	30 (17%)	6 (15%)		
Family history of colorectal cancer in a first‐degree relative						
Absent	1067 (80%)	658 (79%)	376 (81%)	33 (77%)	0.71	0.48
Present	271 (20%)	174 (21%)	87 (19%)	10 (23%)		
Year of diagnosis						
Prior to 1995	466 (35%)	274 (33%)	179 (38%)	13 (30%)	0.28	0.38
1996–2000	424 (31%)	257 (31%)	149 (32%)	18 (42%)		
2001–2018	457 (34%)	306 (36%)	139 (30%)	12 (28%)		
Tumor location						
Cecum	220 (16%)	98 (12%)	109 (23%)	13 (31%)	0.006	0.11
Ascending to transverse colon	415 (31%)	282 (34%)	120 (26%)	13 (31%)		
Distal colon	410 (31%)	261 (31%)	139 (30%)	10 (24%)		
Rectum	295 (22%)	191 (23%)	98 (21%)	6 (14%)		
Tumor differentiation						
Well to moderate	1203 (90%)	725 (87%)	437 (94%)	41 (95%)	0.11	0.72
Poor	137 (10%)	107 (13%)	28 (6%)	2 (5%)		
AJCC disease stage						
I	319 (26%)	212 (28%)	97 (23%)	10 (24%)	0.63	0.37
II	382 (31%)	256 (34%)	113 (27%)	13 (32%)		
III	344 (28%)	199 (26%)	131 (31%)	14 (34%)		
IV	175 (14%)	95 (12%)	76 (18%)	4 (10%)		
CIMP status						
Negative	546 (43%)	335 (43%)	197 (45%)	14 (33%)	0.0005	0.0036
Low	481 (38%)	244 (31%)	215 (49%)	22 (52%)		
High	232 (18%)	198 (25%)	28 (6%)	6 (14%)		
Mean LINE‐1 methylation level (%) ± SD	63.2 ± 10.1	63.6 ± 10.5	62.5 ± 9.1	62.0 ± 9.1	0.3	0.69
LINE‐1 methylation level						
≤50	114 (9%)	69 (9%)	41 (9%)	4 (10%)	0.79	0.97
50‐60	336 (26%)	199 (25%)	125 (27%)	12 (29%)		
>60	840 (65%)	525 (66%)	289 (64%)	26 (62%)		
MSI status						
Non‐MSI‐high	1106 (84%)	633 (77%)	431 (93%)	42 (98%)	0.0016	0.26
MSI‐high	218 (16%)	186 (23%)	31 (7%)	1 (2%)		
*BRAF* mutation						
Wild‐type	1130 (84%)	629 (76%)	459 (98.5%)	42 (100%)	0.0002	0.42
Mutant	211 (16%)	204 (24%)	7 (1.5%)	0 (0%)		
*PIK3CA* mutation						
Wild‐type	1053 (84%)	697 (89%)	328 (76%)	28 (74%)	0.0033	0.7
Mutant	195 (16%)	84 (11%)	101 (24%)	10 (26%)		
*Fusobacterium animalis (nucleatum)*						
Negative	1048 (87%)	374 (87%)	638 (86%)	36 (92%)	0.61	0.47
Low	79 (6%)	30 (7%)	47 (6%)	2 (5%)		
High	82 (7%)	25 (6%)	56 (8%)	1 (3%)		
*pks* ^*+*^ *Escherichia coli*						
Negative	1024 (91%)	359 (90%)	629 (91%)	36 (92%)	0.6	0.86
Low	49 (4%)	14 (4%)	33 (5%)	2 (5%)		
High	55 (5%)	24 (6%)	30 (4%)	1 (3%)		
*Bifidobacterium*						
Negative	874 (70%)	316 (73%)	529 (68%)	29 (69%)	0.65	0.35
Low	207 (17%)	58 (13%)	144 (19%)	5 (12%)		
High	168 (13%)	60 (14%)	100 (13%)	8 (19%)		
*Bacteroides fragilis*						
Negative	552 (50%)	188 (49%)	348 (50%)	16 (44%)	0.83	0.76
Low	277 (25%)	106 (28%)	161 (23%)	10 (28%)		
High	283 (25%)	91 (24%)	182 (26%)	10 (28%)		
Enterotoxigenic *Bacteroides fragilis*						
Negative	1013 (91%)	349 (91%)	629 (91%)	35 (97%)	0.34	0.37
Low	49 (4%)	17 (4%)	31 (5%)	1 (3%)		
High	50 (5%)	19 (5%)	31 (5%)	0 (0%)		

Abbreviations: AJCC, The American Joint Committee on Cancer; CIMP, CpG island methylator phenotype; HPFS, Health Professionals Follow‐up Study; LINE‐1, long‐interspersed nucleotide element‐1; MSI, microsatellite instability; NHS, Nurses’ Health Study; SD, standard deviation.

^a^ Data are presented as number (%) or as the mean ± standard deviation (SD). Percentage indicates the proportion of cases with a specific clinical, pathologic, or molecular characteristic among all colorectal cancer cases or in each stratum of *KRAS* mutation status.

^b^ To compare categorical data by *KRAS* mutation status, the chi‐square test or Fisher's exact test was performed. To compare age and LINE‐1 methylation level by *KRAS* mutation status, the analysis of variance was performed. To compare year of diagnosis by *KRAS* mutation status, AJCC disease stage, and tumour location, the Spearman correlation test was performed.

Notably, the prevalence of CIMP‐low status was similarly higher in *KRAS* c.34G>T (p.G12C) mutants (52%) and other *KRAS* mutants (49%) than in *KRAS*‐wild‐type tumors (31%). In addition, the CIMP category distribution was significantly different in *KRAS* c.34G>T (p.G12C) mutants compared with other *KRAS* mutants (*p* = 0.0036). Specifically, *KRAS* c.34G>T (p.G12C) mutants showed higher CIMP‐high prevalence (14%) and lower CIMP‐negative prevalence (33%) compared with other *KRAS* mutants (6% and 45%, respectively).

Regarding other tumoral features, when compared with *KRAS*‐wild‐type tumors, *KRAS* c.34G>T tumors were more likely to demonstrate cecal location (31% vs. 12%; *p* = 0.006), non‐MSI‐high status (98% vs. 77%; *p* = 0.0016), wild‐type *BRAF* (100% vs. 76%; *p* = 0.0002), and *PIK3CA* mutation (26% vs. 11%; *p* = 0.0033). These associations were similarly present in other *KRAS‐*mutated tumors compared with *KRAS*‐wild‐type tumors. Neither *KRAS* c.34G>T mutants nor other *KRAS* mutants were associated with other features including the tumor tissue abundance of *F. nucleatum* (*F. animalis*), *pks* ^+^ *E. coli*, *Bifidobacterium*, or (enterotoxigenic) *B. fragilis*.

Additionally, because both *KRAS* c.34G>T (p.G12C) mutation and *BRAF* mutation were associated with CIMP‐high status, we conducted exploratory analyses comparing clinical and tumor characteristics between *KRAS* c.34G>T (p.G12C)‐mutated and *BRAF‐*mutated tumors (Table S1). Compared with *BRAF‐*mutated tumors, *KRAS* c.34G>T (p.G12C)‐mutated tumors showed lower proportions of female (51% vs. 76%; *p* = 0.0011), lower poor differentiation prevalence (5% vs. 30%; *p* = 0.0004), lower CIMP‐high prevalence (15% vs. 74%), lower MSI‐high prevalence (2% vs. 58%; *p* < 0.0001), lower *F. nucleatum*‐positivity (8% vs. 23%), lower prevalence of ETBF‐high tumors (0% vs. 10%), and lower mean LINE‐1 methylation level (62.0 vs. 67.7; *p* = 0.0017).

**FIGURE 2:**
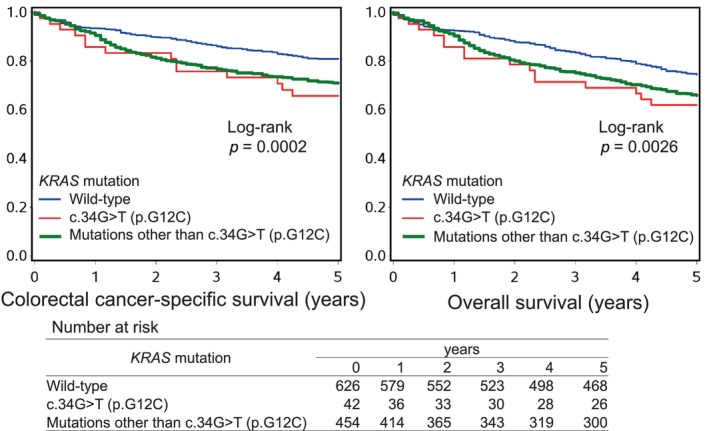
Survival analyses of 1112 *BRAF*‐wild‐type colorectal cancer patients according to the *KRAS* mutation status.

We examined the prognostic role of *KRAS* mutation within 1122 *BRAF*‐wild‐type colorectal carcinoma cases. Among these patients, there were 841 deaths including 347 colorectal cancer‐specific deaths, during a median follow‐up of 20.4 years (interquartile range, 16.2–24.0 years) for censored cases without 5‐year cutoffs. The 5‐year colorectal cancer‐specific survival probabilities were 81% in *KRAS*‐wild‐type patients, 66% in *KRAS* c.34G>T (p.G12C)‐mutated patients, and 71% in other *KRAS*‐mutated patients (Figure 2). We observed significant differences in colorectal cancer‐specific mortality between *KRAS*‐wild‐type, *KRAS* c.34G>T (p.G12C)‐mutated, and other *KRAS‐*mutated patients in Kaplan–Meier analysis (log‐rank *p* = 0.0002; Figure 2). In Cox proportional hazards regression analyses (Table 2), compared with *KRAS*‐wild‐type tumors, *KRAS* c.34G>T (p.G12C)‐mutated tumors were associated with a higher 5‐year colorectal cancer‐specific mortality (with univariable HR, 1.97; 95% confidence interval [CI], 1.13–3.42; *p* = 0.017; and multivariable HR, 1.82; 95% CI, 1.05–3.17; *p* = 0.035). In addition, compared with *KRAS*‐wild‐type tumors, other *KRAS*‐mutated tumors were also associated with a higher 5‐year colorectal cancer‐specific mortality (with univariable HR, 1.63; 95% CI, 1.27–2.09; *p* = 0.0001; and multivariable HR, 1.57; 95% CI, 1.22–2.02; *p* = 0.0004).

**TABLE 2:** Survival analyses of 1112 *BRAF*‐wild‐type colorectal cancer patients according to the *KRAS* mutation status.

*KRAS* mutation status	No. of cases	Colorectal cancer‐specific mortality					Overall mortality				
		No. of events	Univariable HR (95% CI)*	*p*‐Value	Multivariable HR (95% CI)*	*p*‐Value	No. of events	Univariable HR (95% CI)*	*p* value	Multivariable HR (95% CI)*	*p*‐Value
Wild‐type	626	117	1 (referent)		1 (referent)		160	1 (referent)		1 (referent)	
c.34G>T (p.G12C)	42	14	1.97 (1.13–3.42)	0.017	1.82 (1.05–3.17)	0.035	16	1.66 (1.00–2.78)	0.052	1.50 (0.90–2.51)	0.12
Mutations other than c.34G>T (p.G12C)	454	130	1.63 (1.27–2.09)	0.0001	1.57 (1.22–2.02)	0.0004	155	1.43 (1.15–1.78)	0.0016	1.37 (1.10–1.71)	0.0055

Abbreviations: CI, confidence interval; HR, hazard ratio.

* The multivariable Cox regression model initially included age at diagnosis, sex, year of diagnosis, family history of colorectal cancer, tumor location, microsatellite instability, CpG island methylator phenotype, *PIK3CA* mutation, and long interspersed nucleotide element‐1 methylation level. A backward elimination with a threshold of *p* = 0.05 was used to select variables in the final model.

## 4 DISCUSSION

The purpose of this study was to test the hypothesis that certain clinical and tumor characteristics of *KRAS* c.34G>T (p.G12C)‐mutated colorectal carcinomas might differ from those of other *KRAS*‐mutated tumors and *KRAS*‐wild‐type tumors. Compared with other *KRAS*‐mutated tumors, *KRAS* c.34G>T (p.G12C)‐mutated tumors exhibited higher CIMP‐high prevalence and lower CIMP‐negative prevalence. In addition, *KRAS* c.34G>T (p.G12C)‐mutated tumors and other *KRAS*‐mutated tumors similarly showed high CIMP‐low prevalence compared with *KRAS*‐wild‐type tumors. Similar to other *KRAS*‐mutated tumors, *KRAS* c.34G>T (p.G12C)‐mutated tumors were also associated with cecal location, non‐MSI‐high status, *PIK3CA* mutation, and wild‐type *BRAF*. In addition, we showed that, among *BRAF*‐wild‐type cases, both *KRAS* c.34G>T (p.G12C) mutation and *KRAS* mutations other than c.34G>T were associated with higher colorectal cancer‐specific mortality compared with *KRAS*‐wild‐type cases. Hence, our study has provided new intriguing findings on colorectal carcinomas with the specific *KRAS* c.34G>T (p.G12C) mutation.

*KRAS* mutations, which are present in approximately 40% of colorectal carcinomas, have been implicated in tumor development, progression, treatment resistance, and recurrence.^3^ Traditionally, developing targeted therapies for *KRAS* mutations has been challenging due to lack of binding pockets suitable for small‐molecule inhibitors.^34^ Combination therapies with allele‐specific covalent KRAS p.G12C inhibitors and anti‐EGFR targeted therapy have recently shown promising results in advanced colorectal cancer patients with *KRAS* c.34G>T (p.G12C) mutation.^13^, ^14^, ^35^, ^36^ Therefore, it is of particular interest to comprehensively understand the features of *KRAS* c.34G>T (p.G12C)‐mutated colorectal carcinomas.

The clinicopathological features of *KRAS* c.34G>T (p.G12C)‐mutated colorectal carcinomas, in particular, differences from those from other *KRAS‐*mutated carcinomas remain to be characterized. Studies showed that, compared with other *KRAS‐*mutated colorectal cancers, *KRAS* c.34G>T (p.G12C)‐mutated colorectal cancer was associated with male sex, distal colorectal localization, and distant metastases to the lung, liver, and brain.^37^, ^38^, ^39^ In contrast, other studies reported generally similar demographic and clinical characteristics of *KRAS* c.34G>T (p.G12C)‐mutated colorectal cancer to those of other *KRAS*‐mutated colorectal cancers although these studies included only advanced colorectal cancer cases with limited tumor molecular information.^9^, ^19^, ^40^ All of these findings as well as results of the current study need to be replicated in multiple independent studies. We found the unique association of *KRAS* c.34G>T‐mutated colorectal carcinoma with both CIMP‐high (when compared with other *KRAS*‐mutated tumors) and CIMP‐low statuses (when compared with *KRAS*‐wild‐type tumors). The link between *KRAS* mutations and CIMP‐low status in colorectal carcinoma was discovered in the past.^41^ A recent study based on the cohorts and PCITB used in the current study has found a positive relationship of CIMP‐high with the tumor tissue abundance of *B. fragilis*, in particular ETBF,^29^ which has been implicated in colorectal carcinogenesis.^42^ With limited statistical power, we did not observe an association of *KRAS* c.34G>T (p.G12C) mutation with *B. fragilis* or ETBF. CIMP‐high colorectal carcinomas are considered to arise through the serrated neoplasia pathway characterized by serrated morphologies of precursor lesions. Colorectal tumors arising through the serrated neoplasia pathway often carry *BRAF* mutations and only rarely *KRAS* mutations.^43^ Considering our new findings, it is also of interest to study the *KRAS* c.34G>T variant in serrated precursor lesions compared with conventional nonserrated adenomas.

We did not observe a significant association of *KRAS* c.34G>T with the tissue abundance of *F. nucleatum* (*F. animalis*) or *pks* ^*+*^ *E. coli*. These bacteria have been implicated in colorectal carcinogenesis.^44^, ^45^, ^46^, ^47^ Previous studies have shown potential interrelated links between cecal location, *KRAS* mutation, and *F. nucleatum* in colorectal carcinomas.^48^, ^49^, ^50^, ^51^ The relationship between *KRAS* mutations and tissue microorganisms need to be clarified in future studies.

The incidence of early‐onset colorectal cancer has been rising since the 1990s in many parts of the world, which has drawn international attention.^52^, ^53^ We have shown that tumor LINE‐1 hypomethylation is a biomarker of early‐onset colorectal cancer and has been associated with aggressive tumor behaviors.^54^ In this study, compared with *BRAF*‐mutated tumors, *KRAS* c.34G>T mutants showed more frequent LINE‐1 hypomethylation. A possible link of *KRAS* c.34G>T mutation to LINE‐1 hypomethylation and early‐onset carcinoma should be investigated in future larger studies because the sample size of early‐onset carcinoma is limited in this study.

In colorectal cancer, *KRAS* mutation has been reported as an unfavorable prognosis factor,^55^ though the literature data are somewhat mixed likely due at least in part to the presence/absence of the adjustment for a strong confounder variable, *BRAF* mutation. Previous studies demonstrated that the *KRAS* c.34G>T (p.G12C) mutation was associated with poorer prognosis compared with other *KRAS* mutations.^9^, ^37^, ^40^ In our dataset, in addition to the c.34G>T (p.G12C) mutation (shown by the current study), *KRAS* c.34G>C (p.G12R) and c.35G>T (p.G12V) mutations were associated with higher colorectal cancer‐specific mortalities among various *KRAS* mutations.^56^ Therefore, the prognostic impact of various *KRAS* mutations including the c.34G>T (p.G12C) mutation should be investigated by future studies with large sample sizes and detailed tumor molecular and treatment data.

The current study has some limitations. First, the number of *KRAS* c.34G>T (p.G12C)‐mutated cases was modest, as this mutation is present in only 3%–4% of colorectal cancer patients. Second, the participants in our study consisted of health professionals and predominantly non‐Hispanic white individuals. Therefore, our findings should be replicated in different populations. Third, in survival analyses, cancer treatment data were not available in most cases. Nonetheless, our findings of the prognostic role of the *KRAS* c.34G>T (p.G12C) mutation are consistent with prior studies by other investigators.

The current study has notable strengths. First, we conducted robust CIMP analyses using the rigorously validated bisulfite modification and MethyLight assay with high sensitivity and specificity to detect CIMP‐high status using FFPE tumor tissue.^24^, ^26^ Second, we assembled tumor tissue materials of colorectal carcinoma patients (in the PCITB within the well‐defined cohort populations) from hundreds of hospitals located throughout the United States, which increases the generalizability of study findings compared with a study based on a limited number of hospitals. Third, based on two large‐scale prospective cohort studies, our molecular pathological epidemiology database could assess the features of *KRAS* c.34G>T‐mutated colorectal cancer, including the prognostic role of this specific *KRAS* c.34G>T mutation independent of *BRAF* mutation and many other various clinicopathological and tumoral features.

In conclusion, this study provides novel evidence for clinical and tumor characteristics of *KRAS* c.34G>T (p.G12C)‐mutated colorectal carcinoma, which needs to be replicated by independent datasets. Given that KRAS p.G12C protein variant inhibitors can be an effective treatment strategy for advanced colorectal cancer, our study is likely of clinical relevance.

Use of standardized official symbols: We use Human Genome Organisation (HUGO) Gene Nomenclature Committee (HGNC)‐approved official symbols (or root symbols), accompanied by unique HGNC ID where appropriate, for genes and gene products, including BRAF, CACNA1G, CDKN2A, CRABP1, EGFR, IGF2, KRAS, MAPK, MLH1, NEUROG1, PIK3CA, RAS, RUNX3, and SOCS1, all of which are described at www.genenames.org. The official gene symbols are italicized to differentiate from nonitalicized gene product names and nonofficial colloquial names.

## AUTHOR CONTRIBUTIONS

**Satoko Ugai:** Formal analysis; funding acquisition; investigation; project administration; writing – original draft; writing – review and editing. **Qian Yao:** Writing – review and editing. **Yasutoshi Takashima:** Writing – review and editing. **Yuxue Zhong:** Writing – review and editing. **Kosuke Matsuda:** Writing – review and editing. **Hidetaka Kawamura:** Writing – review and editing. **Yu Imamura:** Writing – review and editing. **Kazuo Okadome:** Writing – review and editing. **Kosuke Mima:** Writing – review and editing. **Kota Arima:** Writing – review and editing. **Keisuke Kosumi:** Writing – review and editing. **Mingyang Song:** Writing – review and editing. **Jeffrey A. Meyerhardt:** Writing – review and editing. **Marios Giannakis:** Writing – review and editing. **Jonathan A. Nowak:** Writing – review and editing. **Tomotaka Ugai:** Formal analysis; funding acquisition; investigation; methodology; project administration; resources; supervision; writing – original draft; writing – review and editing. **Shuji Ogino:** Conceptualization; formal analysis; investigation; methodology; project administration; resources; supervision; validation; writing – original draft; writing – review and editing.

## FUNDING INFORMATION

This work was supported by US National Institutes of Health (NIH) grants (P01 CA87969; UM1 CA186107; P01 CA55075; UM1 CA167552; U01 CA167552; R35 CA197735 to S.O.; R01 CA151993 to S.O.; R21 CA230873 to S.O.; and R50 CA274122 to T.U.); by Cancer Research UK Grand Challenge Award (C10674/A27140 to M.G. and S.O.). The content is solely the responsibility of the authors and does not necessarily represent the official views of NIH. T.U. was supported by Prevent Cancer Foundation grant, Harvey V. Fineberg Cancer Prevention Fellowship, Brigham and Women's Hospital Faculty Career Development Award, an investigator‐initiated grant from the American Institute for Cancer Research (AICR). Each of K.M., H.K., T.U., and S.U. was separately supported by a fellowship grant from the Uehara Memorial Foundation. S.O. is an American Cancer Society Clinical Research Professor (CRP‐24‐1185864‐01‐PROF). The funders had no role in study design, data collection and analysis, decision to publish, or preparation of the manuscript.

## CONFLICT OF INTEREST STATEMENT

M.G. receives research funding from Janssen, consulting fees from Nerviano Medical Sciences, and has received Honoraria from AstraZeneca and Chroma Code. No other conflicts of interest exist. The other authors have no conflicts of interest.

## ETHICS STATEMENT

Approval of the research protocol by an Institutional Reviewer Board: The study protocol was approved by the institutional review boards of the Brigham and Women's Hospital and Harvard T.H. Chan School of Public Health, and those of participating registries as required.

Informed Consent: Informed consent was obtained from all of the subjects.

Registry and the Registration No. of the study/trial: N/A.

Animal Studies: N/A.

## Supporting information


Table S1.

## Data Availability

Further information including the procedures to obtain and access data from the Nurses' Health Studies and Health Professionals Follow‐up Study is described at https://www.nurseshealthstudy.org/researchers (contact email: nhsaccess@channing.harvard.edu) and https://sites.sph.harvard.edu/hpfs/for‐collaborators/.
