# Supplementary material for: Clinicopathological, molecular, and prognostic features of colorectal carcinomas with KRAS c.34G>T (p.G12C) mutation
Source: Cancer Sci. 2024 Jul 22;115(10):3455–65. doi: 10.1111/cas.16262 (PMC11448363; doi:10.1111/cas.16262)
Supplement: Supplementary file 1 — Table S1. [file CAS-115-3455-s001.docx]

**Table S1.** Clinicopathological, Molecular, and Microbial Characteristics of *KRAS* c.34G>T (p.G12C) Mutant Cases and *BRAF* Mutant Cases.

|  | *KRAS* c.34G>T (p.G12C) | *BRAF* mutant | P value |
| --- | --- | --- | --- |
|  | (N = 43) | (N = 211) |  |
| Sex |  |  | 0.0011 |
| Male (HPFS) | 21 (49%) | 50 (24%) |  |
| Female (NHS) | 22 (51%) | 161 (76%) |  |
| Mean age ± SD (years) | 70.3±9.2 | 70.7±8.1 | 0.73 |
| Cumulative pack-years smoked |  |  | 0.15 |
| 0 | 20 (50%) | 75 (37%) |  |
| 1-19 | 10 (25%) | 39 (19%) |  |
| 20-39 | 4 (10%) | 34 (17%) |  |
| ≥40 | 6 (15%) | 55 (27%) |  |
| Family history of colorectal cancer in a first-degree relative |  |  | 0.84 |
| Absent | 33 (77%) | 165 (78%) |  |
| Present | 10 (23%) | 46 (22%) |  |
| Year of diagnosis |  |  | 0.10 |
| Prior to 1995 | 13 (30%) | 52 (25%) |  |
| 1996-2000 | 18 (42%) | 67 (32%) |  |
| 2001-2018 | 12 (28%) | 92 (44%) |  |
| Tumor location |  |  | 0.81 |
| Cecum | 13 (31%) | 27 (13%) |  |
| Ascending to transverse colon | 13 (31%) | 145 (69%) |  |
| Distal colon | 10 (24%) | 28 (13%) |  |
| Rectum | 6 (14%) | 9 (4%) |  |
| Tumor differentiation |  |  | 0.0004 |
| Well to moderate | 41 (95%) | 148 (70%) |  |
| Poor | 2 (5%) | 63 (30%) |  |
| AJCC disease stage |  |  | 0.52 |
| I | 10 (24%) | 37 (18%) |  |
| II | 13 (32%) | 83 (41%) |  |
| III | 14 (34%) | 47 (23%) |  |
| IV | 4 (10%) | 36 (18%) |  |
| CIMP status |  |  | <0.0001 |
| Negative | 13 (32%) | 11 (5%) |  |
| Low | 22 (54%) | 42 (21%) |  |
| High | 6 (15%) | 149 (74%) |  |
| Mean LINE-1 methylation level (%) ± SD | 62.0±9.0 | 67.7±10.3 | 0.0017 |
| LINE-1 methylation level |  |  | 0.077 |
| ≤50 | 4 (10%) | 10 (5%) |  |
| 50-60 | 11 (27%) | 31 (15%) |  |
| >60 | 26 (63%) | 161 (80%) |  |
| MSI status |  |  | <0.0001 |
| Non-MSI-high | 42 (98%) | 88 (42%) |  |
| MSI-high | 1 (2%) | 121 (58%) |  |
| *PIK3CA* mutation |  |  | 0.067 |
| Wild-type | 28 (74%) | 170 (86%) |  |
| Mutant | 10 (26%) | 28 (14%) |  |
| *Fusobacterium animalis (nucleatum)* |  |  | 0.15 |
| Negative | 35 (92%) | 154 (77%) |  |
| Low | 2 (5%) | 18 (9%) |  |
| High | 1 (3%) | 27 (14%) |  |
| *pks^+^ Escherichia coli* |  |  | 0.78 |
| Negative | 36 (92%) | 163 (92%) |  |
| Low | 2 (5%) | 9 (5%) |  |
| High | 1 (3%) | 6 (3%) |  |
| *Bifidobacterium* |  |  | 0.46 |
| Negative | 29 (69%) | 135 (66%) |  |
| Low | 5 (12%) | 36 (18%) |  |
| High | 8 (19%) | 34 (17%) |  |
| *Bacteroides fragilis* |  |  | 0.20 |
| Negative | 16 (44%) | 89 (47%) |  |
| Low | 10 (28%) | 29 (15%) |  |
| High | 10 (28%) | 70 (37%) |  |
| Enterotoxigenic *Bacteroides fragilis* |  |  | 0.20 |
| Negative | 35 (97%) | 164 (87%) |  |
| Low | 1 (3%) | 5 (3%) |  |
| High | 0 (0%) | 19 (10%) |  |

* Data are presented as number (%) or as the mean ± standard deviation (SD). Percentage indicates the proportion of cases with a specific clinical, pathologic, or molecular characteristic among all colorectal cancer cases or in each stratum of *KRAS* mutation status.

† To compare categorical data by *KRAS* mutation status, the chi-square test or Fisher’s exact test was performed. To compare age and LINE-1 methylation level by those status, the analysis of variance was performed. To compare year of diagnosis by those levels, AJCC disease stage, and tumour location, the Spearman correlation test was performed.

Abbreviations: AJCC, The American Joint Committee on Cancer; CIMP, CpG island methylator phenotype; HPFS, Health Professionals Follow-up Study; LINE-1, long-interspersed nucleotide element-1; MSI, microsatellite instability; NHS, Nurses’ Health Study; SD, standard deviation.
